# Logistic regression and other statistical tools in diagnostic biomarker studies

**DOI:** 10.1007/s12094-024-03413-8

**Published:** 2024-03-26

**Authors:** Dina Mohamed Ahmed Samir Elkahwagy, Caroline Joseph Kiriacos, Manar Mansour

**Affiliations:** Pharmaceutical Biology Department, Faculty of Pharmacy and Biotechnology, German University in Cairo, Cairo, 11835 Egypt

**Keywords:** Biomarker, Statistics, Diagnostic, Logistic regression, Receiving operatic characteristic, ROC, Cutoff

## Abstract

A biomarker is a measured indicator of a variety of processes, and is often used as a clinical tool for the diagnosis of diseases. While the developmental process of biomarkers from lab to clinic is complex, initial exploratory stages often focus on characterizing the potential of biomarkers through utilizing various statistical methods that can be used to assess their discriminatory performance, establish an appropriate cut-off that transforms continuous data to apt binary responses of confirming or excluding a diagnosis, or establish a robust association when tested against confounders. This review aims to provide a gentle introduction to the most common tools found in diagnostic biomarker studies used to assess the performance of biomarkers with an emphasis on logistic regression.

## Background

According to the Biomarkers consortium National Institute of Health (NIH), biomarkers are parameters that are objectively measured and evaluated as indicators of normal biological processes, pathogenic processes, or pharmacologic responses to therapeutic intervention. Biomarkers may be generally classified according to their use as indicated in Table 1 [1].

**Table 1:** Types of biomarkers

Type	Definition
Diagnostic	Used to determine or verify if a disease or other condition is present or not
Prognostic	To determine the likelihood of disease recurrence or outcome in relation to the levels of the biomarker
Predictive	To determine the association of an effect in relation to the levels of the biomarker when exposed to a therapeutic agent or environmental factor
Monitoring	Measured periodically to determine the severity of an illness or medical condition, as well as to determine if a therapeutic agent or environmental factor has had any impact
Risk	To determine the likelihood of an effect or disease in relation to the levels of the biomarker
Response	To determine whether a therapeutic agent or environmental factor has had any biological effect

### Different types of biomarkers require different characterizations

**Fig. 1:**
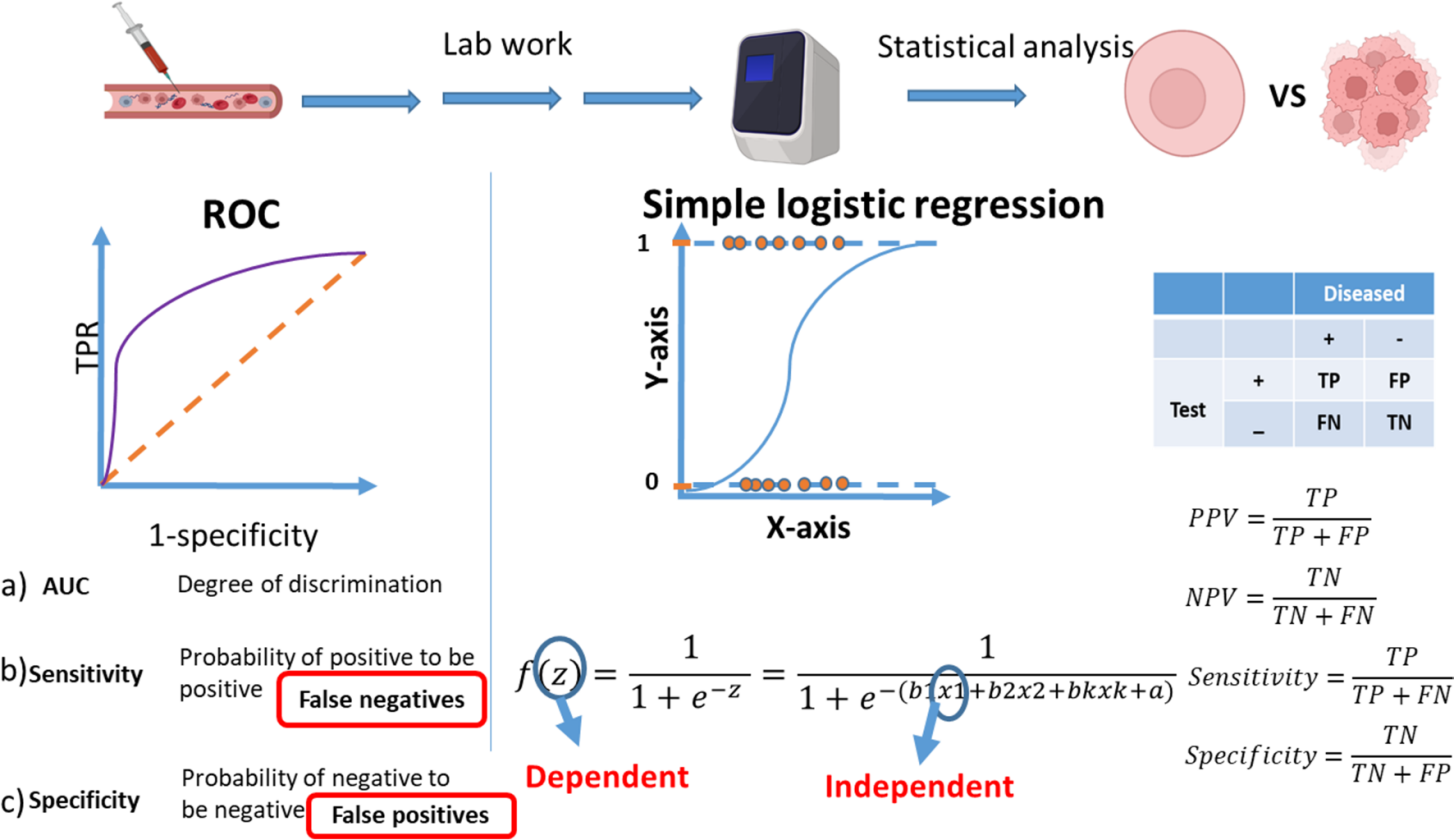
Key statistical tools for plasma biomarkers as an example. This figure sheds the light on an example for the main statistical tools used in plasma biomarker research. The results obtained from the qPCR are analyzed using statistical tools as ROC. ROC is a plot between the true positive rate and false positive rate. AUC is calculated with its corresponding *p* value. Thus, it can determine the marker’s ability to discriminate between patients and controls. Sensitivity and specificity are calculated as well. A prediction model is conducted also to predict a variable by one or more other variables and measure the influence of one or more variable on another variable. PPV, NPV, sensitivity and specificity were also calculated from the regression model

The process of biomarker development comprises five phases based on the Early Detection Research Network, with each phase building upon the results of the previous one [2]. These phases are arranged according to the strength of evidence, progressing from weaker to stronger. Statistical tests are conducted in each phase to determine significance. However, the discussed tests are most critical in the initial phases. Figure 1 highlights an example for using different statistical tools in biomarker research from plasma.

These phases begin with discovery and progress through analytical validation, clinical validation or biological validation, clinical utility, and eventually, the final stage of associated implementation factors such as legal, ethical, and social ramifications as well as cost effectiveness.

A biomarker needs to meet a few fundamental requirements before it can move on to the discovery phase/first phase. It must be readily available, simple to prepare and store, and available in sufficient quantities to meet its measurement requirements.

Analytical validation (the second phase) involves assessing the reproducibility of the biomarker measurements. Variables such as cut-off values, limits of detection, linearity, accuracy and precision, sensitivity and specificity, inter- and intra-assay coefficients of variation, and other factors are assessed at this stage.

The focus of the third stage, clinical validation, is the evaluation of qualities built on the thresholds established from the previous two phases. These performance indicators include likelihood and hazard ratios, area under the curve (AUC) or receiver operating characteristic curve (ROC), sensitivity and specificity, and positive and negative predictive values.

Any biomarker’s ultimate objective is to make it through the fourth most difficult stage of clinical utility. The performance of a marker is finally decided at this stage because it will be the basis for further clinical decisions. Because of this, not all markers deemed trustworthy or accurate may be ultimately accepted [3]. Assessing what qualifies a biomarker as clinically helpful is crucial and starts with quantifying the diagnostic properties; therefore, the main factors and specifications that must be met for diagnostic tests to be considered of clinical interest are covered in the next section.

Biomarker discovery and development occurs over various steps of qualification and validation that is supported by various statistical elements that ensure reproducibility and utility of the biomarker within its context of use [4, 5]. Initial stages of establishing a link between the biomarker and the disease’s outcome are supplemented by statistical tools that quantify the relationship to not only provide further degrees of evidence of its performance, but also enable tailoring of the biomarker for its intended use [6]. For example, diseases with low prevalence rates would benefit more from biomarkers having higher specificity rather than sensitivity [7]. Establishing preliminary characteristics of performance in initial stages additionally helps guide future directions within the study [8].

## Criteria for a useful diagnostic test

The traditional method of testing the usefulness or accuracy of a diagnostic test is to measure it against a reference diagnosis typically used in clinical settings.

Diagnostic tests are often binary in their conclusion: they either aim to confirm or exclude a diagnosis. While many statistical methods exist, certain measures of diagnostic accuracy are more commonly used than others to characterize biomarkers. Such measures include classification probabilities (true positive fraction or TPF/sensitivity, true negative fraction or TNF/specificity), predictive values (positive predictive value or PPV, negative predictive value or NPV), diagnostic odds ratios (DORs), likelihood ratios (LRs), ROC curves, and Euclidean and Youden indexes. While some measures are discriminative (for example, ROC curves), others could be predictive (as in the case with logistic regression) in nature. Predictive measures are most helpful in determining the likelihood that a disease will afflict an individual, for example, while discriminative measures are typically used to simply classify those with the disease from those without. While good discriminative performance is often more aligned with diagnostic biomarkers, predictive measures are helpful in quantifying the magnitude of the test’s result on the outcome. The ideal diagnostic biomarker would be one to discriminate perfectly, being able to completely diagnose an individual with a disease without any false diagnoses taking place. However, it is often difficult to realize such concepts for a variety of reasons. The choice on the acceptable degree of diagnostic uncertainty would then be based on a variety of factors on the clinical level, such as the nature of the disease, the cost of medical care, and the psychological effects of a missed diagnosis.

### Sensitivity and specificity

Sensitivity (the test’s ability to truly detect all people with the disease, or the true positive) and specificity (the test’s ability to discount all people without the disease) are common metrics used to assess a diagnostic test. Although a test with both high sensitivity and specificity is desirable, trade-offs can be made depending on the intent of use, setting and the nature of the disease itself to prioritize one over the other. Sensitivity and specificity can be derived by simple equations from a confusion matrix (also known as a classification table), as demonstrated in Table 2. It includes all possible possibilities in a clinical setting: true positive indicates those correctly diagnosed with the disease, false positives are those diagnosed without actually having the disease, false negative indicates those misdiagnosed as healthy despite actually having the disease, and finally true negatives are those correctly diagnosed as not having the disease [9]. All the equations derived from the matrix are shown in Table 3.

**Table 2:** Confusion matrix or classification table

	Observed	
	True	False
*Predicted*		
True	True Positive (TP)	False Positive (FP)
False	False Negative (FN)	True Negative (TN)

**Table 3:** Diagnostic equations derived from the confusion matrix

Possibilities in clinical setting	Equation
False positive rate (FPR)	FP/FP + TN
False negative rate (FNR)	FN/FN + TP
Sensitivity	TP/FN + TP
Specificity	TN/TN + FP
Accuracy	TN + TP/TN + FP + FN + TP

The implications of false positives and negatives should be considered when designing the metrics and cut-offs of the diagnostic test. For example, a false negative means that a patient is misleadingly thought to be healthy until further symptoms develop or mortality occurs as a result of no treatment. Such a consequence is made worse in diseases where early diagnosis could result in treatment and full recovery or a better prognosis at minimum. On the other hand, a false positive result would cause unnecessary, if not harmful, medical interventions that may cause financial, psychological and overall avoidable harm to the individual.

Certain aspects of the disease are also critical in designing and evaluating diagnostic tests, particularly disease prevalence. Prevalence is defined as the fraction of people in the population having the disease as opposed to the total population under study itself. Prevalence is an important characteristic to take into consideration, particularly in metrics of diagnostic accuracy such as predictive values [10].

The tradeoffs in the measures of accuracy are therefore evaluated by assessing the relative risk of false positive or negative results within the population of the disease while taking into account the prevalence of the disease within the population itself as well.

### PPV and NPV

The positive predictive value is the proportion of correctly predicted cases with the observed outcome versus the total number of cases predicted to have the outcome. The negative predictive value, on the other hand, is the proportion of correctly predicted cases lacking the observed characteristic in comparison with the overall number of cases predicted as not having the outcome. PPV and NPV are functions of prevalence and are influenced by prevalence. In other words, to calculate the two values, the prevalence must be known. While PPV and NPV are metrics often used in diagnostic accuracy studies, any interpretation derived would not be generalizable across studies, as they are greatly affected by prevalence. Meaning the interpretation derived would only be exclusive to the studied population.

In general, high specificity (ability to correctly diagnose those without disease or false positives/true negatives) tends to occur with a high PPV (ratio of truly diagnosed over all the diagnosed) value due to the presence of few false positives/falsely diagnosed.

### ROC curve

The receiver operating characteristic curve is a curve drawn by joining together a series of points obtained from the determination of (sensitivity/true positive; 1—specificity/false positive) at different cut-offs. The area under the generated curve is used to evaluate classification performance with all possible different cut-offs of the biomarker.

There is no absolute consensus or calculation to derive what would be an acceptable AUC for a diagnostic biomarker, but generally speaking, most studies tend to follow general guideline values highlighted in Table 4 to evaluate the value calculated by the plot [11, 12]: Greater AUC values indicate better test performance, with AUC values that can range from 0.5 (no diagnostic ability) to 1.0. (Perfect diagnostic ability). The ROC curve is an important statistical technique for evaluating the performance of diagnostic medical tests, especially for tests that aim to detect cancers early [13].

**Table 4:** General interpretation of AUC values

AUC of ROC	Interpretation
0.50–0.60	No value diagnostic biomarker
0.60–0.70	Poor diagnostic biomarker
0.70–0.80	Acceptable diagnostic biomarker
0.80–0.90	Good diagnostic biomarker
0.90–1.00	Excellent diagnostic biomarker

Another way of interpretation would be to take into consideration the clinical setting where the biomarker will be used to determine whether the given AUC would have any meaningful significance.

### Logistic regression

To fit models for the probability of disease as the outcome given marker values, logistic regression is used. Logistic regression, also known as the logistic model or the logit model, examines the relationship between a single or several independent continuous variables and a dichotomous/binary dependent variable. These types of analyses create a model to relate the outcome (the dependent variable), to the predictor variable (the independent variable). The probability of the occurrence of an outcome is estimated by fitting input data from epidemiological data (for example, patients and controls) to a logistic curve, where the predictive power is represented as the regression coefficients. There are two types of models used in analyses, depending on the number of possible outcomes in the dependent (Predictor) variable: if it is two/dichotomous, then binary logistic regression is utilized, and if it consists of more than two then multivariate logistic regression is used. Possible uses of logistic regression in the field of biomarker studies are highlighted in Table 5.

**Table 5:** Possible uses of logistic regression is diagnostic studies

Application	Response variable	Predictor variable	References
Association	Has septic shock or not?	Expression value of 13 lncRNAs from Microarray sets	[32]
	High and low MSC-AS1 levels	The clinicopathological features of gastric cancer	[33]
Predictive model	clinically diagnosed Pulmonary tuberculosis (PTB) cases/patients with suspected PTB	LncRNA levels and electronic health records	[34]
	Has septic shock/normal controls	LncRNA levels	[35]
Screening test (risk score analysis)	Risk of developing severe acute pancreatitis	systemic inflammatory response syndrome, albumin, blood urea nitrogen and pleural effusion	[36]
	Predicting hilar cholangiocarcinoma development	Levels of lncRNA	[37]
Risk assessment	Metabolic syndrome	Various Biomarkers	[38]
Adjusting for confounder effects	1. Plasma or CSF biomarker levels (beta-amyloid, phosphorylated tau, neurofilament light, glial fibrillary acidic protein) 2. development of dementia	1. Plasma or CSF biomarkers (beta-amyloid, phosphorylated tau, neurofilament light, glial fibrillary acidic protein) with confounders 2. Plasma biomarkers and confounders	[39]

Feature selection is another aspect of logistic regression that may be beneficial in the early stages of biomarker discovery, especially in high throughput techniques (for example, “-omics” methods involving DNA or RNA sequencing, or mass spectrometry) [14], where many potential candidates exist. Such methods help decrease the dimensionality of the data by removing redundant or irrelevant candidates to minimize complexity and further fine-tune the model generated to prevent overfitting [15]. This can be performed through several broad methods that include filter, wrapper, and embedded methods. The methods are classified depending on whether or not a model needs to be generated through learning algorithms like logistic regression in order to assess the features, with filter being the only methodology out of the three to act independently of the model [16]. Hybrid methods that combine two or all three exists as well [17]. An overview of each method’s strengths and weaknesses is highlighted in Table 6.

**Table 6:** The advantages and disadvantages of each feature selection method that is commonly used with learning algorithms

Methods	Advantages	Disadvantages	References
Filter	1. Less computationally exhaustive 2. Faster 3. Less risk of overfitting/more generalizable due to being independent of model	1. decreased predictive performance of model 2. Due to generalizability, often selects a large number (if not all) of features	[40–42]
Embedded	1. Selects features to cater better to the model, with both feature selection and model training done simultaneously 2. Performs better than filter method	1. Risk of overfitting 2. Slower than filter method Not free from model’s bias due to depending on it 3. Computationally exhaustive	[40–42]
Wrapper	1. Selects features to cater better to the model 2. Performs better than filter method	1. Risk of overfitting 2. Not free from model’s bias due to depending on it 3. Slowest method 4. Most computationally exhaustive, least favorable with high volume datasets	[40–42]

The evaluation of the logistic regression model includes multiple phases. The overall model is evaluated in terms of the relationship between all independent variables and the dependent variable. Then, the significance of the independent variable or variables is determined by assessing the derived regression coefficient per variable. Another phase includes assessing the model’s predictive accuracy/discriminating ability. The model must then be validated. The exhaustive steps are underlined below:

1. *Evaluation of the overall model*
  The overall fit of a model can be evaluated by comparing the predicted model to a null model (a model with no independent variable) when fitted to the input data. The model is said to be a better fit only if it exhibits improvement over the empty model [18], which is usually assessed through an Omnibus test or a Hosmer & Lemeshow test [11, 19].
2. *Predictive accuracy and discrimination of model*
  Once the fitness of a model is evaluated, the accuracy is assessed. The accuracy can be determined from the sensitivity and specificity of the model, which is calculated using a confusion matrix. A user defined cut-off is defined by the user (anywhere from 0 to 1) where all predicted values above the cut-off are classified as predictive [18].
3. *Statistical significance of regression coefficients of independent variable*
  Is the predictive power of the independent variable significant enough? The relationship between the dependent and independent variable can be confirmed through statistical significance, which can be assessed by multiple tests such as the Wald statistic, the odds ratio, and the likelihood ratio test [18, 20].
4. *Validation of the model*
  Once the model has been constructed, one final point must be assessed: whether the model developed with the independent/predictor variables can correctly predict the dependent/outcome variable in another subset of the population. There are two major methods of validation: external and internal. External validation is performed by testing the model on an entirely different dataset than the one used to build the model. Internal validation is performed using a similar subset of the population used to develop the model, if not the same.
  4.a *Validation by frequentist approach*
  The split-sample technique is performed by randomly splitting the dataset into training and validation sets. The disadvantages of such a method include the reduction of the dataset sample size used to develop the model, and different splitting formats may produce different results. Cross-validation mimics the split-sample method of dividing the sample into a training and validation set but adds to it in that it is a resampling technique where development and testing are done in rounds.
  Another commonly used method is bootstrap validation. This type depends on a hypothetical test set created based on the given values and is used to validate the model. In bootstrapping, the complete dataset is resampled several times with replacement, with statistics being generated on each resampling, and the statistics from each resampling are merged in a specific way. In logistic regression models developed in smaller samples, bootstrapping is commonly used to derive optimal estimates of internal validity [21].
  Biomarker studies that have been published with logistic regression often report either the coefficient of the logistic regression equation or the odds ratio (which is simply the exponent of the coefficient) [22], along with the confidence intervals (CI) or the significance (*p* value), to indicate the statistical significance of the associations established by these values between the predictor variable and the outcome variable.

### Bayesian approach

The Bayesian approach is another statistical language approach that can substitute conventional logistic regression. This language has the ability to take into consideration our beliefs (current beliefs) and obtain the probability of distribution. The following equation demonstrates Bayes’ theorem.

This approach depends mainly on the availability of prior probabilities before conducting the study which is represented as P(A) (probability of A occurring). P(B/A) is the probability of event B to occur given A and this is termed the likelihood. P(B) is the probability of B to occur, and this is termed the evidence. Finally, from all this information, Pr(A/B) is computed, which is the posterior distribution, meaning that the prior is converted to posterior after taking into account the results of the experiment [23].

One main advantage in this approach is its ability to validate the model if the data available is limited. For instance, rare diseases could be a hurdle that face any clinical study due to small number of patients in the population [24, 25]. It also gives a range for how to be certain for or against a hypothesis rather than a point estimate. However, it is still a more complex type of statistical analysis, and more advanced statistical software is needed to utilize this method.

One main disadvantage, on the other hand, is that priors could be subjective and possibly affect the posterior distribution in some way. Moreover, the presence of priors is critical, which is not possible without the analysis.

### Cut-off determination

In diagnostic studies, the test should yield binary outcomes (positive or negative). When a new biomarker is explored, the optimum cut-off to transform the continuous values into dichotomous ones is assessed through the use of several metrics that often incorporate sensitivity and specificity [26]. A general outline is detailed below of the most common calculations used for such assessments.

### Youden’s index

An optimum cut-off in the statistical sense would be one with the greatest possible difference between the total positive rate (i.e., Sensitivity) and false positive rate (i.e., 1-Specificity) [27].

### Diagnostic odds ratios/DOR

The DOR of a test is the ratio of the odds of positivity in diseased subjects compared to the odds of positivity in healthy subjects. The ratio is derived from sensitivity and specificity and as a result, is not affected by the prevalence of the disease [28]. DOR can be calculated using the following equation:

$$\begin{aligned} {\text{DOR}} & = & \left( {{\text{Sensitivity}}*{\text{Specificity}}} \right)/\left( {1 - {\text{Specificity}}/{\text{False positives}}*1 - {\text{Sensitivity}}/{\text{False negatives}}} \right) \, \\ \quad {\text{or }}\left( {{\text{Sensitivity}}/1 - {\text{Sensitivity}}} \right)/\left( {1 - {\text{Specificity}}/{\text{Specificity}}} \right). \\ \end{aligned}$$

Values higher than one generally indicate some degree of diagnostic usefulness [28], with increasing values indicating better performances. The DOR is commonly used as a measure of association in epidemiology; however, the discriminatory power is often put to the question [29, 30]. Since an odds ratio is a single number, it does not account for the trade-off between accurately identifying cancer patients and mistakenly identifying otherwise healthy individuals, but may be useful in characterizing population level risks [29]. Hence, some studies discourage the use of DOR when examining binary early detection biomarkers [31].

### Likelihood ratios/LR

It is defined as the ratio of the probability of correctly diagnosing the disease in patients with the target disease to the probability of incorrectly diagnosing the disease. The LR predicts how likely a patient would have a disease using sensitivity and specificity. (LR+ indicates positive test results, while LR- indicates negative test results).

They are calculated using the following equations:

$${\text{LR}}\, + \, = {\text{Sensitivity}}/1 - {\text{Specificity}}$$

$${\text{LR}} - = 1 - {\text{Sensitivity}}/{\text{Specificity}}$$

Rough guidelines on how LR is generally interpreted in the literature [27] are highlighted in Table 7**.**

**Table 7:** General interpretations of LR values

LR	Interpretation
>10	Large and often conclusive increase in the likelihood of disease
5–10	Moderate increase in the likelihood of disease
2–5	Small increase in the likelihood of disease
1–2	Minimal increase in the likelihood of disease
1	No change in the likelihood of disease
0.5–1.0	Minimal decrease in the likelihood of disease
0.2–0.5	Small decrease in the likelihood of disease
0.1–0.2	Moderate decrease in the likelihood of disease
<0.1	Large and often conclusive decrease in the likelihood of disease

## Conclusion

The clinical field is still in an immense need for the development of new biomarkers.

Biomarkers offer guidance for clinicians at the beginning or throughout the clinical intervention itself. They could be screening, diagnostic, prognostic, predictive, monitoring, risk or response. Regardless of their specific use, studying biomarkers is often tied to statistical analysis. Statistical analyses are often carried out by biostatisticians.

The hurdles encountered by clinical researchers in statistical analysis are often attributed to the lack of a comprehensive and straightforward guide outlining the essential steps, together with their corresponding definitions, calculation methods, and reasoning behind why and how each calculation is used. This review serves as a general guide for the main statistical analyses that are needed to develop and validate a biomarker study.

## Data Availability

The datasets generated during and/or analysed during the current study are available from the corresponding author upon reasonable request.

## Abbreviations

NIH: National Institute of Health
EDRN: Early Detection Research Network
AUC: Area under the curve
ROC: Receiver operating characteristics curve
TPF: True positive fraction
TNF: True negative fraction
PPV: Positive predictive value
NPV: Negative predictive value
DOR: Diagnostic odds ratio
LR: Likelihood ratio
CI: Confidence interval
PTB: Pulmonary tuberculosis

## References

[1] Califf RM. Biomarker definitions and their applications. Exp Biol Med (Maywood). 2018;243:213–21. 29405771 10.1177/1535370217750088 PMC5813875

[2] Five-Phase approach and prospective specimen collection, retrospective blinded evaluation study design [Internet]. Early Detection Research Network. [cited 2023 Nov 17]. https://edrn.nci.nih.gov/about-edrn/five-phase-approach-and-prospective-specimen-collection-retrospective-blinded-evaluation-study-design/.

[3] Dobbin KK, Cesano A, Alvarez J, Hawtin R, Janetzki S, Kirsch I, et al. Validation of biomarkers to predict response to immunotherapy in cancer: Volume II—clinical validation and regulatory considerations. J Immunother Cancer. 2016;4:77. 10.1186/s40425-016-0179-0 PMC5109653 27891226

[4] Kraus VB, Blanco FJ, Englund M, Henrotin Y, Lohmander LS, Losina E, et al. OARSI clinical trials recommendations: soluble biomarker assessments in clinical trials in osteoarthritis. Osteoarthr Cartil. 2015;23:686–97. 10.1016/j.joca.2015.03.002 PMC4430113 25952342

[5] Gosho M, Nagashima K, Sato Y. Study Designs and statistical analyses for biomarker research. Sensors (Basel). 2012;12:8966–86. 23012528 10.3390/s120708966 PMC3444086

[6] Parikh CR, Philbrook HT. Chapter 2—statistical considerations in analysis and interpretation of biomarker studies. In: Edelstein CL, editor. Biomarkers of kidney disease [Internet]. San Diego: Academic Press; 2011 [cited 2024 Feb 14]. p. 25–37. https://www.sciencedirect.com/science/article/pii/B9780123756725100027.

[7] Chen R, Crispin DA, Pan S, Hawley S, McIntosh MW, May D, et al. Pilot study of blood biomarker candidates for detection of pancreatic cancer. Pancreas. 2010;39:981–8. 20467349 10.1097/MPA.0b013e3181dac920 PMC4060618

[8] Yee LM, Lively TG, McShane LM. Biomarkers in early-phase trials: fundamental issues. Bioanalysis. 2018;10:933–44. 29923753 10.4155/bio-2018-0006 PMC6123886

[9] Trevethan R. Sensitivity, specificity, and predictive values: foundations, pliabilities, and pitfalls in research and practice. Front Public Health. 2017;5:307. 29209603 10.3389/fpubh.2017.00307 PMC5701930

[10] Šimundić A-M. Measures of diagnostic accuracy: basic definitions. EJIFCC. 2009;19:203–11. 27683318 PMC4975285

[11] Hosmer DW, Lemeshow S, Sturdivant RX. Applied logistic regression. 3rd ed. Hoboken, NJ: Wiley; 2013.

[12] Metz CE. Basic principles of ROC analysis. Semin Nucl Med. 1978;8:283–98. 112681 10.1016/S0001-2998(78)80014-2

[13] Baker SG. The central role of receiver operating characteristic (ROC) curves in evaluating tests for the early detection of cancer. JNCI J Natl Cancer Inst. 2003;95:511–5. 12671018 10.1093/jnci/95.7.511

[14] Szymczak S, Biernacka JM, Cordell HJ, González-Recio O, König IR, Zhang H, et al. Machine learning in genome-wide association studies. Genet Epidemiol. 2009;33:S51–7. 19924717 10.1002/gepi.20473

[15] Yu L, Liu H. Eficient Feature Selection Via Analysis of Relevance and Redundancy. J Mach Learn Res. 2004;5:1205–24.

[16] Bolón-Canedo V, Sánchez-Maroño N, Alonso-Betanzos A. A review of feature selection methods on synthetic data. Knowl Inf Syst. 2013;34:483–519. 10.1007/s10115-012-0487-8

[17] Remeseiro B, Bolon-Canedo V. A review of feature selection methods in medical applications. Comput Biol Med. 2019;112:103375. 31382212 10.1016/j.compbiomed.2019.103375

[18] Park H-A. An introduction to logistic regression: from basic concepts to interpretation with particular attention to nursing domain. J Korean Acad Nurs. 2013;43:154. 23703593 10.4040/jkan.2013.43.2.154

[19] Hosmer DW, Hosmer T, Le Cessie S, Lemeshow S. A comparison of goodness-of-fit tests for the logistic regression model. Stat Med. 1997;16:965–80. 9160492 10.1002/(SICI)1097-0258(19970515)16:9<965::AID-SIM509>3.0.CO;2-O

[20] Harrell FE. Regression modeling strategies. Bios. 2017;330:14.

[21] Arboretti Giancristofaro R, Salmaso L. Model performance analysis and model validation in logistic regression. Statistica. 2003; 63, 2007(2):375396.

[22] Weber DG, Casjens S, Johnen G, Bryk O, Raiko I, Pesch B, et al. Combination of MiR-103a-3p and mesothelin improves the biomarker performance of malignant mesothelioma diagnosis. Altomare DA, editor. PLoS ONE. 2014;9:e114483. 10.1371/journal.pone.0114483 PMC4255020 25469901

[23] Furukawa K, Ohyama T. The Bayesian approach to evidence-based decision making. J Hepatobiliary Pancreat Sci. 2021;28:457–60. 34028193 10.1002/jhbp.997

[24] Garczarek U, Muehlemann N, Richard F, Yajnik P, Russek-Cohen E. Bayesian strategies in rare diseases. Ther Innov Regul Sci. 2023;57:445–52. 36566312 10.1007/s43441-022-00485-y PMC9789883

[25] van de Schoot R, Broere JJ, Perryck KH, Zondervan-Zwijnenburg M, van Loey NE. Analyzing small data sets using Bayesian estimation: the case of posttraumatic stress symptoms following mechanical ventilation in burn survivors. Eur J Psychotraumatol. 2015;6:25216. 25765534 10.3402/ejpt.v6.25216 PMC4357639

[26] Hajian-Tilaki K. The choice of methods in determining the optimal cut-off value for quantitative diagnostic test evaluation. Stat Methods Med Res. 2018;27:2374–83. 28673124 10.1177/0962280216680383

[27] Parikh CR, Thiessen Philbrook H. Statistical considerations in analysis and interpretation of biomarker studies. biomarkers of kidney disease [Internet]. Elsevier; 2017 [cited 2023 Apr 21]. p. 21–32. https://linkinghub.elsevier.com/retrieve/pii/B9780128030141000029.

[28] Glas AS, Lijmer JG, Prins MH, Bonsel GJ, Bossuyt PMM. The diagnostic odds ratio: a single indicator of test performance. J Clin Epidemiol. 2003;56:1129–35. 14615004 10.1016/S0895-4356(03)00177-X

[29] Pepe MS, Janes H, Longton G, Leisenring W, Newcomb P. Limitations of the odds ratio in gauging the performance of a diagnostic, prognostic, or screening marker. Am J Epidemiol. 2004;159:882–90. 15105181 10.1093/aje/kwh101

[30] Böhning D, Holling H, Patilea V. A limitation of the diagnostic-odds ratio in determining an optimal cut-off value for a continuous diagnostic test. Stat Methods Med Res. 2011;20:541–50. 20639268 10.1177/0962280210374532

[31] Baker SG, Kramer BS, Srivastava S. Markers for early detection of cancer: statistical guidelines for nested case-control studies. BMC Med Res Methodol. 2002;2:4. 11914137 10.1186/1471-2288-2-4 PMC100327

[32] Zheng X, Leung K-S, Wong M-H, Cheng L. Long non-coding RNA pairs to assist in diagnosing sepsis. BMC Genomics. 2021;22:275. 33863291 10.1186/s12864-021-07576-4 PMC8050902

[33] Yang W, Ge F, Lu S, Shan Z, Peng L, Chai J, et al. LncRNA MSC-AS1 Is a diagnostic biomarker and predicts poor prognosis in patients with gastric cancer by integrated bioinformatics analysis. Front Med (Lausanne). 2021;8:795427. 34926534 10.3389/fmed.2021.795427 PMC8674534

[34] Hu X, Liao S, Bai H, Gupta S, Zhou Y, Zhou J, et al. Long noncoding RNA and predictive model to improve diagnosis of clinically diagnosed pulmonary tuberculosis. Land GA, editor. J Clin Microbiol. 2020;58:e01973–19. 10.1128/JCM.01973-19 PMC7315016 32295893

[35] Wu Y, Yin Q, Zhang X, Zhu P, Luan H, Chen Y. Long noncoding RNA THAP9-AS1 and TSPOAP1-AS1 provide potential diagnostic signatures for pediatric septic shock. Biomed Res Int. 2020;2020:7170464. 33344646 10.1155/2020/7170464 PMC7725549

[36] Hong W, Lillemoe KD, Pan S, Zimmer V, Kontopantelis E, Stock S, et al. Development and validation of a risk prediction score for severe acute pancreatitis. J Transl Med. 2019;17:146. 31068202 10.1186/s12967-019-1903-6 PMC6505180

[37] Shi J, Li X, Zhang F, Kong L, Zhang X, Cheng Y, et al. The plasma LncRNA acting as fingerprint in hilar cholangiocarcinoma. Cell Physiol Biochem. 2018;49:1694–702. 30231247 10.1159/000493613

[38] Xie Q-Y, Wang M-W, Hu Z-Y, Cao C-J, Wang C, Kang J-Y, et al. Screening the influence of biomarkers for metabolic syndrome in occupational population based on the lasso algorithm. Front Public Health. 2021;9:743731. 34712642 10.3389/fpubh.2021.743731 PMC8545799

[39] Pichet Binette A, Janelidze S, Cullen N, Dage JL, Bateman RJ, Zetterberg H, et al. Confounding factors of Alzheimer’s disease plasma biomarkers and their impact on clinical performance. Alzheimer’s & Dementia. 2023;19:1403–14. 10.1002/alz.12787 PMC10499000 36152307

[40] Pudjihartono N, Fadason T, Kempa-Liehr AW, O’Sullivan JM. A Review of feature selection methods for machine learning-based disease risk prediction. Front Bioinform. 2022;2:927312. 36304293 10.3389/fbinf.2022.927312 PMC9580915

[41] Sánchez-Maroño N, Alonso-Betanzos A, Tombilla-Sanromán M. Filter methods for feature selection—a comparative study. In: Yin H, Tino P, Corchado E, Byrne W, Yao X, editors. Intelligent data engineering and automated learning—IDEAL 2007 [Internet]. Berlin, Heidelberg: Springer Berlin Heidelberg; 2007 [cited 2024 Feb 14]. p. 178–87. 10.1007/978-3-540-77226-2_19.

[42] Aziz R, Verma CK, Srivastava N, Department of Mathematics & Computer Application, Maulana Azad National Institute of Technology Bhopal-462003 (M.P.) India. Dimension reduction methods for microarray data: a review. AIMS Bioeng. 2017;4:179–97.

