# Correction: Logistic regression and other statistical tools in diagnostic biomarker studies

**DOI:** 10.1007/s12094-024-03545-x

**Published:** 2024-07-01

**Authors:** Dina Mohamed Ahmed Samir Elkahwagy, Caroline Joseph Kiriacos, Manar Mansour

**Affiliations:** Pharmaceutical Biology Department, Faculty of Pharmacy and Biotechnology, German University in Cairo, Cairo, 11835 Egypt

**Correction: Clinical and Translational Oncology** 10.1007/s12094-024-03413-8

In this article Manar Mansour at affiliation ‘Pharmaceutical Biology Department, Faculty of Pharmacy and Biotechnology, German University in Cairo, Cairo 11835, Egypt’ was missing from the author list. 

 In this article the contribution statement for Manar Mansour was missing and should have been ‘MM contributed to the conception and critical revision of major parts of the manuscript’.

 In the author’s contribution section, the sentence starting ‘The final version of the manuscript...’ was incorrectly given as ‘The final version of the manuscript was approved and revised by both authors’ but should have been ‘The final version of the manuscript was revised and approved by all authors’.

The original article has been corrected.

